# Environmental sound classification using temporal-frequency attention based convolutional neural network

**DOI:** 10.1038/s41598-021-01045-4

**Published:** 2021-11-03

**Authors:** Wenjie Mu, Bo Yin, Xianqing Huang, Jiali Xu, Zehua Du

**Affiliations:** 1 College of Information Science and Engineering, Ocean University of China, Qingdao, China; 2 Pilot National Laboratory for Marine Science and Technology, Qingdao, China

**Keywords:** Electrical and electronic engineering, Acoustics

## Abstract

Environmental sound classification is one of the important issues in the audio recognition field. Compared with structured sounds such as speech and music, the time–frequency structure of environmental sounds is more complicated. In order to learn time and frequency features from Log-Mel spectrogram more effectively, a temporal-frequency attention based convolutional neural network model (TFCNN) is proposed in this paper. Firstly, an experiment that is used as motivation in proposed method is designed to verify the effect of a specific frequency band in the spectrogram on model classification. Secondly, two new attention mechanisms, temporal attention mechanism and frequency attention mechanism, are proposed. These mechanisms can focus on key frequency bands and semantic related time frames on the spectrogram to reduce the influence of background noise and irrelevant frequency bands. Then, a feature information complementarity is formed by combining these mechanisms to more accurately capture the critical time–frequency features. In such a way, the representation ability of the network model can be greatly improved. Finally, experiments on two public data sets, UrbanSound 8 K and ESC-50, demonstrate the effectiveness of the proposed method.

## Introduction

In recent years, the research on environmental sound classification (ESC), which is dedicated mainly to identify specific sound events, such as identifying dog barking, gunshots, and air conditioning sounds, has received increasing attention. The study result has been used in many practical applications, including robotic hearing^1^, smart home^2^, audio monitoring system^3^ soundscape assessment^4^ and so on. Compared with regular and structured sounds such as speech and music, the environmental sound has neither static time patterns like melodies or rhythms nor semantic sequences like phonemes. Hence, it is difficult to find universal features that can represent various temporal patterns. Besides, the environmental sound contains a lot of noise and some sounds unrelated to the sound event, which lead to complicated composition structure with variability, diversity, and unstructured characteristics.

To deal with the above problems, various signal processing methods and machine learning techniques have been used for ESC tasks. In traditional ESC methods^5–7^, appropriate feature representation and efficient classification model are usually regarded as two separate problems. Most of the methods are first to make appropriate feature representations through manual operation, including the Mel frequency cepstral coefficient (MFCC)^8^, Mel spectrum feature^9^, and wavelet transforms^10^. Then, machine learning algorithms such as support vector machines (SVM)^11^, K-nearest neighbors (KNN)^12^, matrix factorization^13^ and extreme learning machines^14^ are used to deal with the generated features. Although these methods improve recognition performance to a certain extent, they also have obvious shortcomings. It takes a lot of time to construct feature representations through manual operation, and to find the best combination of functions, a lot of experiments are often required, the process is very cumbersome. However, with the development of deep learning theory, deep neural networks have been proven to have a strong ability to automatically extract features, making more deep network models^15–18^ used to solve the ESC problem. In particular, the convolution neural network (CNN) has been proved to have a strong ability to capture time–frequency features^19,20^, which can perfectly solve the limitations of traditional methods, so it is considered very suitable for solving ESC tasks.

Recently, research based on attention mechanism has also been applied to related fields of audio recognition, including speech recognition^20^ and speech sentiment analysis^22^. In the field of ESC, there have also been documents that have proposed a classification model based on the attention mechanism^23–25^. By using neural network to predict the importance of each time step and assigned corresponding weights to each time step based on the prediction results, achieved better performance on some public datasets. However, as the spectrogram is a two-dimensional signal representation of time and frequency, its features in the time–frequency domain have different nature and importance. Although the audio signal conversion in time domain does not have much effect on model classification, the difference between across frequency bands in the frequency domain will greatly affect the classification performance. The above studies only used the attention mechanism to focus on feature vectors at different time steps, but ignored the importance of features in different frequency bands.

Hence, an experiment is designed to analyze the frequency band characteristics to obtain more insights about the influence of different frequency bands on the model classification. After that, a new frequency attention mechanism is then proposed to pay different degrees of attention to each frequency band, so as to focus on learning the feature representation with distinguishing information. Finally, by combining with other temporal attention mechanisms, a novel temporal-frequency attention based convolutional neural network model (TFCNN) is proposed. The model has strong representation ability, and can more effectively capture the critical time–frequency features in sound events. Experiments on the UrbanSound8K and ESC-50 dataset show that the accuracy of the proposed classification model is 93.1% and 84.4%, respectively, which fully proves the advanced nature of the proposed method.

The rest of this paper is set up as follows. Section 2 discusses and reviews previous related work. Section 3 analyzes the frequency band characteristics through experiments, and use as the motivation for the proposed frequency attention mechanism. Section 4 introduces our proposed classification model architecture and attention mechanism. Section 5 reports and analyzes the experimental results. Section 6 summarizes the full text.

## Related work

### ESC networks

This sub-section focuses on the development of deep learning theory utilized in the ESC field. Piczak^19^ first applied the CNN model to solve the ESC problem, and utilized Log-Mel and its deltas spectrogram as a two-dimensional feature representation to input into the network for learning and classification. Compared with the previous traditional methods, the performance has been significantly improved. The modeling capabilities of deep neural networks often require a huge amount of data as support. To solve the problem of the scarcity of labeled environmental sound data, Salamon^26^ proposed several data augmentation strategies. Time stretching, adding background noise, pitch shift, and other means to form new training samples. Compared with the method proposed by Piczak^19^, its accuracy is improved by 6%. Dai^27^ et al. used utilized the original audio waveform as input to train CNN, and conducted a large number of experimental comparisons with the number of CNN layers as the independent variable, the results show that when the number of CNN layers reaches 18 layers, its performance can compete with 2D-CNN using two-dimensional spectrogram as feature input. In^28^, Abdoli et al. proposed an end-to-end classification model based on 1D-CNN, which can directly extract features from raw audio waveforms of any length. They initialized the first layer of the 1D-CNN model as a Gammatone filter bank to simulate the response mechanism of human hearing, which can achieve an accuracy of up to 89%, which is the best performance of the current 1D-CNN model.

### Attention mechanism

The attention mechanism was first proposed in the field of image recognition^29^, mainly used to improve the effect of Encoder and Decoder based on RNN model. In recent years, with the deepening of deep learning research, by combining the attention mechanism with the deep neural network, the importance metric is calculated through the neural network and automatically assigned the corresponding weight for each frame-level feature, breakthroughs have been made in the fields of speech recognition^21^ and machine translation^30^.

To further improve the classification performance of the model, the research based on the attention mechanism has also been carried out in the field of ESC. Guo^23^ et al. first proposed an temporal attention mechanism and extended it to the CLDNN model. The mechanism can evaluate each time step in an attempt to find the most critical time step in the sequence and assign it a higher weight score. Li^24^ et al. proposed a multi-stream network model based on temporal attention. The attention weight is calculated by the degree of energy change in the spectrogram. In^25^, Zhang et al. considered that not all frame-level features can contribute equally to the performance of environmental sound. Except for time frames related to semantic features, others such as silent frames, noise frames, etc. both will reduce the robustness of the classification model and lead to classification errors. Based on this assumption, a frame-level temporal attention mechanism is proposed and extended it to the RNN model to capture the most important time frame part of the sound sequence. Although the above methods improve the classification performance to some extent, the impact of different frequency bands on classification is ignored. Therefore, this paper proposes a frequency attention mechanism that can give different degrees of attention to each frequency band. Combined with temporal attention mechanism, the environmental sound spectrogram with complex time–frequency structures could be well processed.

## Analysis of frequency band characteristics

**Figure 1:**
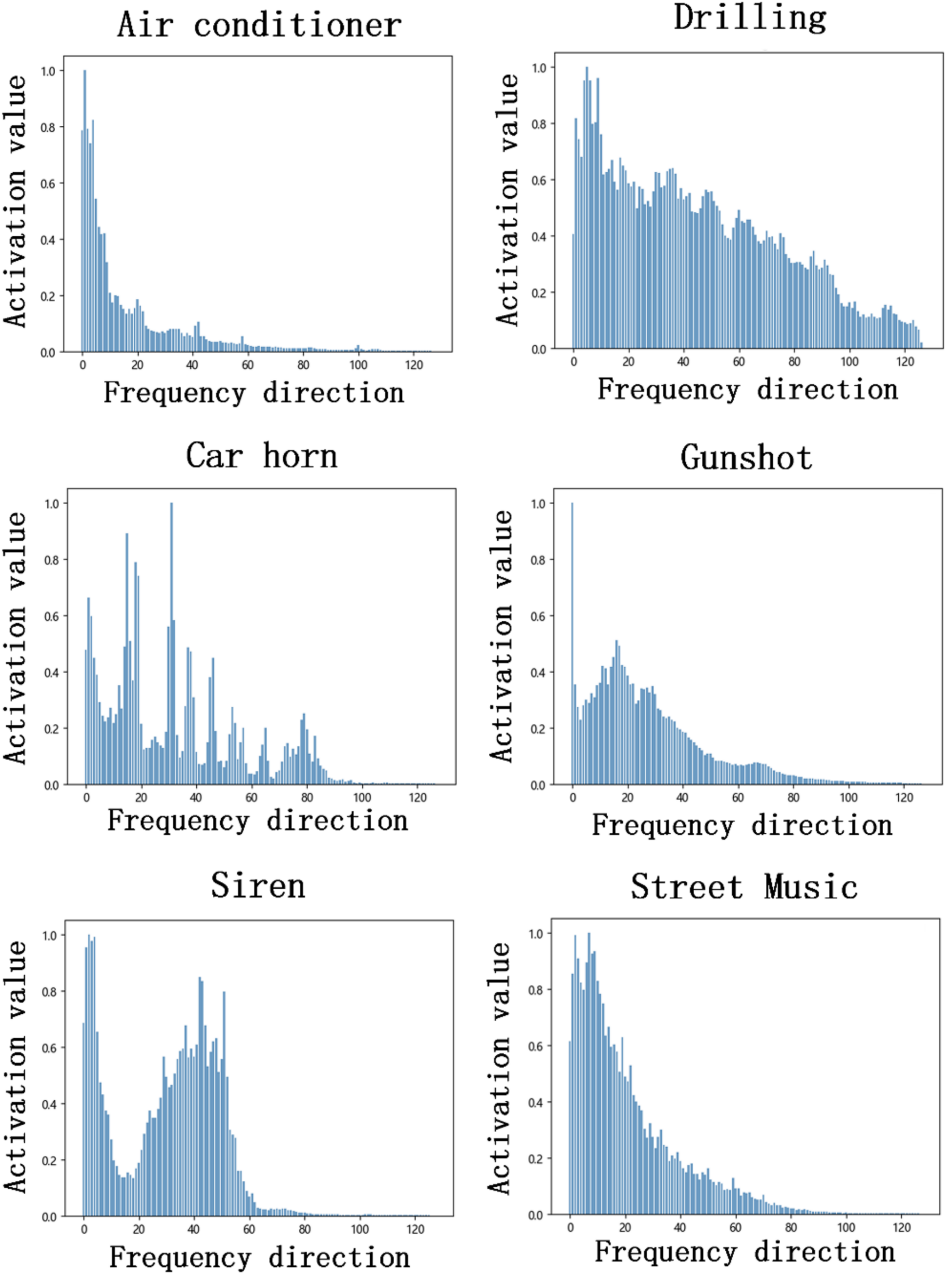
Frequency activation matrix for different sound categories.

To clarify the response of each frequency band on the spectrogram and the impact on the model classification, we designed the following experiment. We first resample the original audio samples at 22050 Hz, use a Hamming window with a size of 1024, and a hop length of 512 to perform a short-time Fourier transform (STFT) on the down-sampled data to extract the amplitude spectrogram. Then, the amplitude spectrogram is passed through 128-Mel filter bank of bands and converted to a logarithmic scale to obtain a Log-Mel spectrogram. After normalizing the Log-Mel spectrogram, connect all samples of the same type on the UrbanSound8k dataset are connected in time dimension, and take the average value in the time direction to obtain 10 frequency activation matrices **X** of size (128, 1), as shown in Fig. 1. It can be observed that the activation values of different Mel frequency bands in each category have obvious changes, and the active frequency bands with higher activation value between different categories are also not exactly. For example, the active band of the “car horn” is relatively scattered and lacks continuity, while the active band of the “siren” is mainly concentrated in the middle and low frequency regions. For the category of “drilling”, there is a higher activation in almost all the frequency bands.

**Figure 2:**
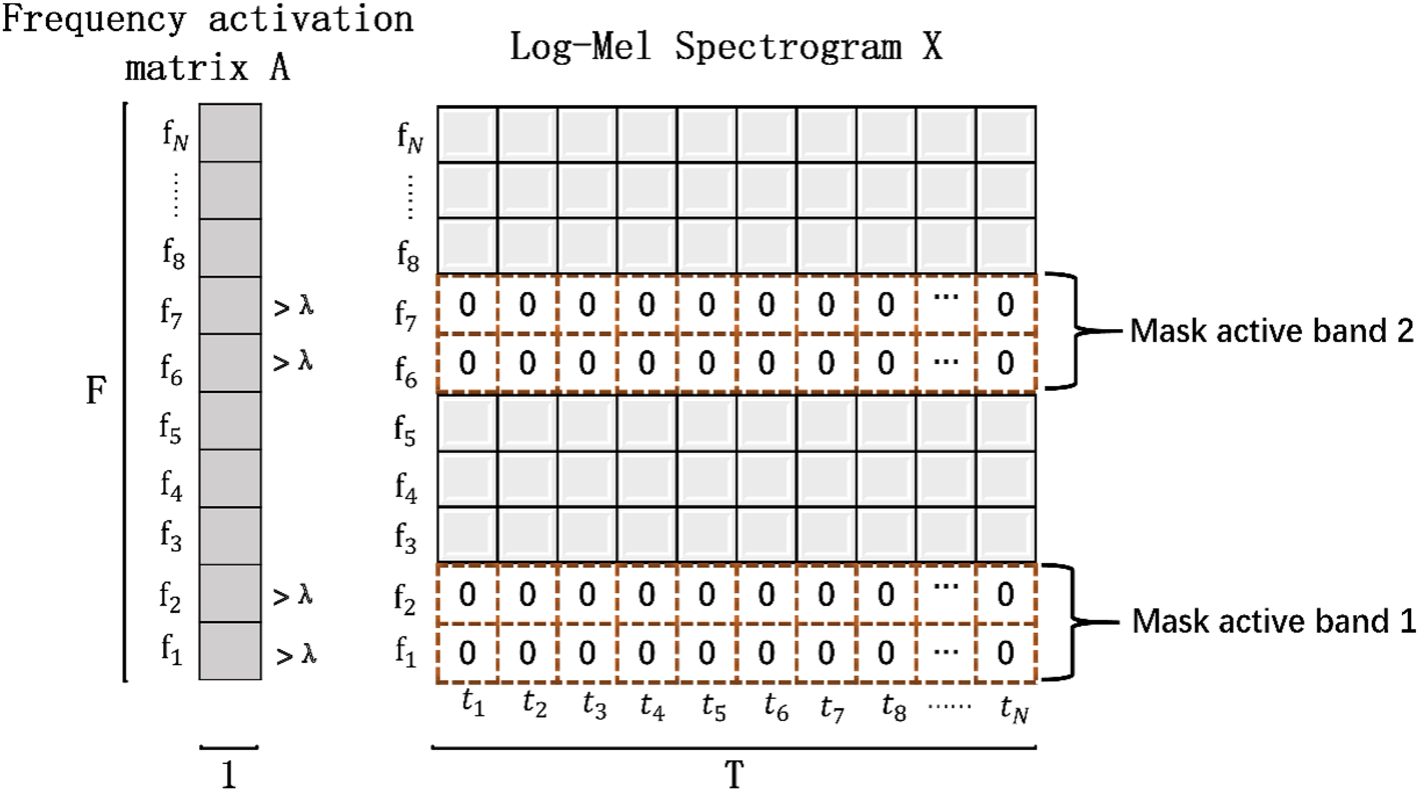
Mask the two active frequency bands of length 2 in the Log-Mel Spectrogram.


In order to analyze how the frequency bands with different activation values affect the classification, we design a method to mask the specific frequency bands (Algorithm 1). The masking effect of this method is shown in Fig. 2. Given the Log-Mel spectrogram **X** of a sample and the frequency activation matrix **A** of the sample category, one or more continuous segments of length **l** active or inactive frequency bands can be masked. We assume that the activation value on the activation matrix A of a sample is greater than λ, then the frequency band corresponding to the position is considered to be an active frequency bands, otherwise it is regarded as an inactive frequency band. ${\mathbf{M}}_{\mathbf{N}}$ is the number of masks.

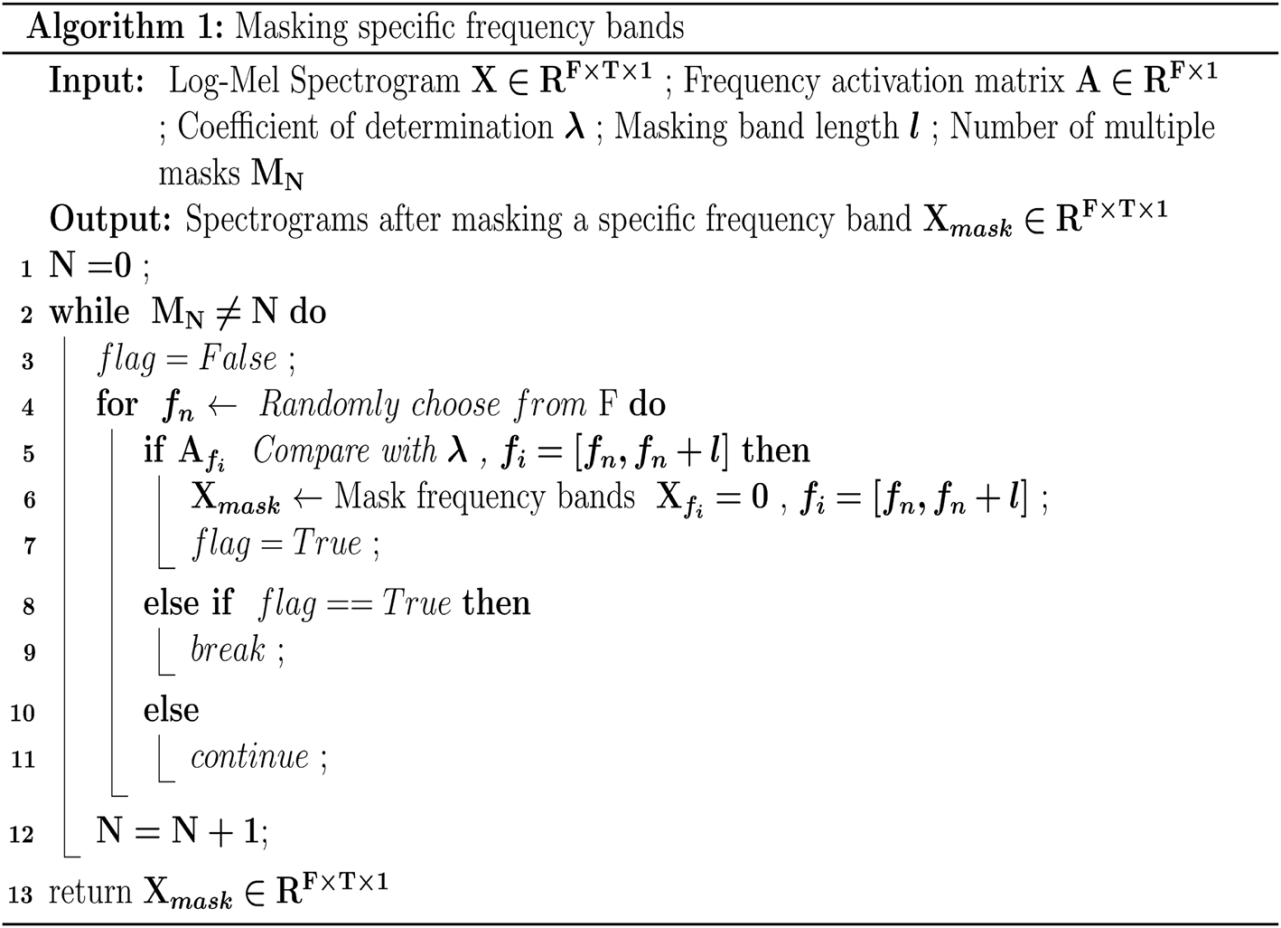


As can be seen from Fig. 1, since the active frequency bands of different types of sound events are basically different, this is not only reflected in the length and position, but also in the number. Corresponding to the three elements of position, length, and quantity, respectively, are the three parameters λ, **l**, and ${\mathbf{M}}_{\mathbf{N}}$ in algorithm 1, so they need to be set according to the specific conditions of each class. Refer to the parameters shown in Table 1, we perform active frequency band masking and inactive frequency band masking for each audio sample in the UrbanSound8k dataset, and construct two datasets according to the different masking strategies–-masking the active frequency band dataset and mask the inactive frequency band dataset. The experimental results are shown in Table 2. As expected, the classification accuracy of the model trained with the masked active frequency band dataset is only 78.3%, which is significantly lower than that of the original dataset for almost all categories. In contrast, the classification accuracy of the model trained with the masked inactive frequency band data set dropped by 3.4%, but it can still maintain most of the performance. Although the artificial definition of active frequency band will inevitably lead to some deviation, but through the above experiments, it can still be explained that those active frequency bands with high activation values will be more important than other frequency bands and contain key distinguishing information that can be used to represent the main activity of the sound event. Moreover, because the essence of the attention mechanism tends to focus on key information that is distinguishable and ignores irrelevant information, this also provides a feasible basis for the frequency attention mechanism we propose next.

**Table 1:** Masking parameters for different categories.

	**AC**	**CH**	**CP**	**DB**	**DR**	**EI**	**GS**	**JA**	**SI**	**SM**
**Λ**	0.1	0.2	0.2	0.3	0.4	0.1	0.2	0.2	0.3	0.4
${\mathbf{M}}_{\mathbf{N}}$	1	2	1	2	1	1	1	1	2	1
**L**	16	15/4	18	18/6	30	15	20	16	20/8	18

**Table 2:** Classification accuracy after training with different datasets.

Class	No mask	Mask I	Mask A
AC	91.5%	90.0%	77.2%
CH	90.9%	88.3%	82.8%
CP	86.3%	82.1%	75.5%
DB	88.0%	85.2%	76.6%
DR	90.1%	82.9%	72.8%
EI	92.8%	93.9%	76.9%
GS	94.7%	91.2%	84.9%
JA	91.0%	85.6%	80.6%
SI	93.3%	90.7%	79.4%
SM	89.1%	84.7%	76.6%
Ave	90.8%	87.4%	78.3%

## Proposed method

**Figure 3:**
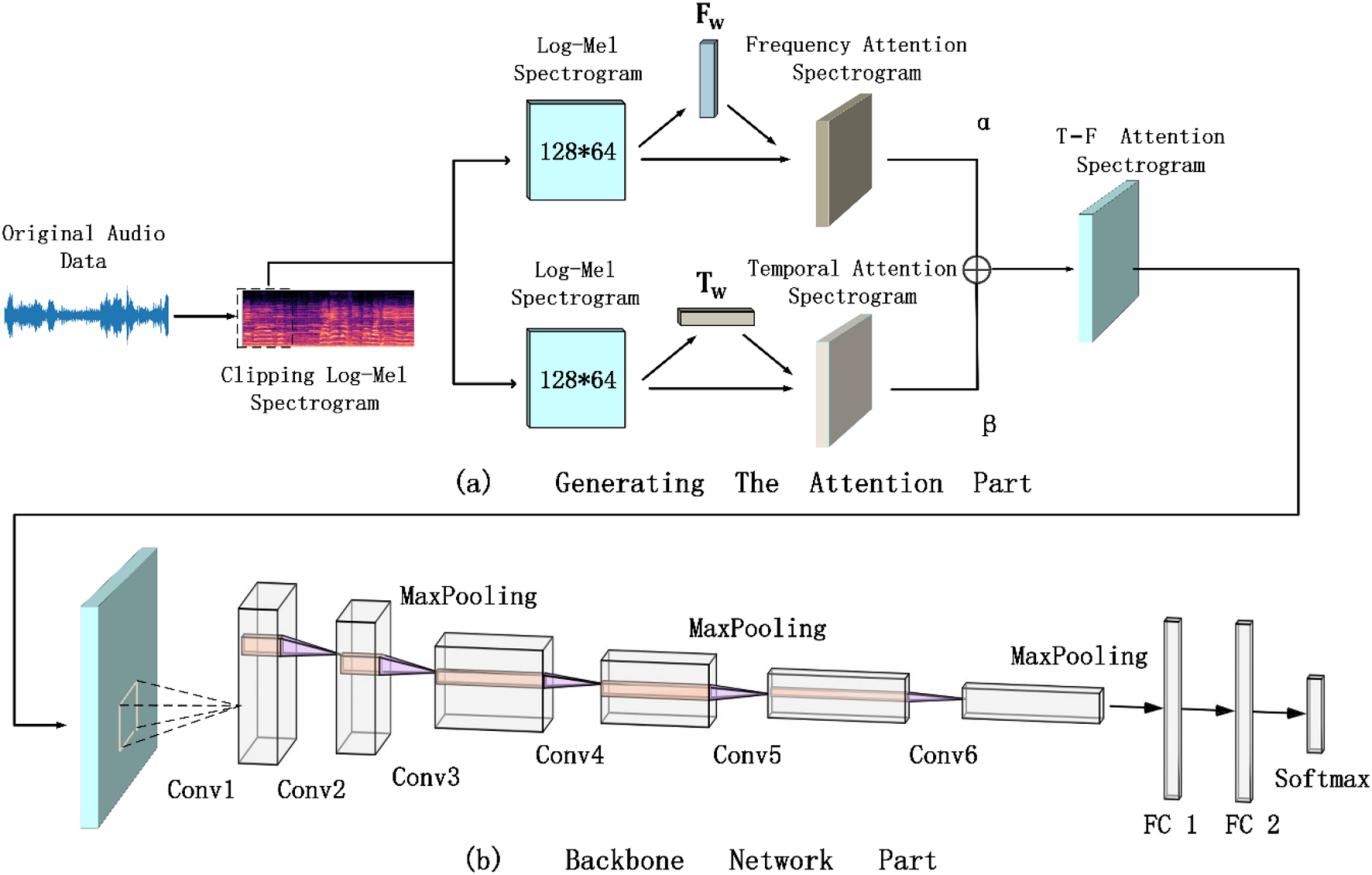
The overall architecture of the proposed TFCNN classification model.

In order to better learn the discriminatory time–frequency features from the audio spectrogram, a temporal-frequency attention baesd convolutional neural network (TFCNN) is proposed in this paper. The overall architecture of the model is shown in Fig. 3, which mainly consists of two parts: generating the attention part and backbone network part. In the part of generating the attention mechanism, by applying the two attention mechanisms developed to the Log-Mel spectrogram extracted from the original audio data, different degrees of attention can be given to the frequency band and time frame parts, so that the calculations used to representation learning can be concentrated in specific areas. The backbone network part consists of a convolutional layer, a pooling layer and a fully connected layer, which is responsible for extracting time–frequency features from the spectrogram processed by attention mechanism and predicting sound events. Besides, in the final testing stage, a probabilistic voting strategy is adopted to summarize the prediction results of multiple audio clips to make judgments, which can effectively avoid classification errors caused by some extreme values.

### Feature processing

In the field of audio recognition, the Log-Mel spectrogram is generally regarded as one of the most powerful features due to the consideration of the human auditory mechanism, the two-dimensional feature map generated by the log-Mel feature along each frame of the audio sequence contains time and frequency features respectively in time domain and frequency domain. Therefore, this paper focuses on using Log-Mel spectrogram as a basic feature to learn the time–frequency representation of environmental sound events, and use CNN in a similar way to image recognition to accurately classify it.

The original audio data format should first be unified and then converted into mono form using average double channel mode. Next, a Hamming window with a size of 46 ms (1024frams, sampling rate 22050 Hz) and overlap of 50\% is used to perform a short-time Fourier transform (STFT) on the data to extract the amplitude spectrogram. After that, the amplitude spectrogram is passed through 128-Mel filter bank of bands and converted to a logarithmic scale to obtain a Log-Mel spectrogram. In the previous literature^25,26,37–41^, the segmentation length is usually set to 41, 44 and 128. However, these values are not suitable for the attention mechanism to learn the importance weight and remain the number of training samples here. Therefore, the Log-Mel spectrogram is split into 64 frames with 50% overlap, and use the zero-padding method to complete the sub-segments with the length less than 64 frames. Finally, the Log-Mel feature of each sub-segment can be expressed as a feature vector of size 128 × 64 × 1 (corresponding to frequency × time × channel).

### Harmonic-percussive source separation

In previous studies, the purpose of the Harmonic-Percussive Source Separation (HPSS) algorithm was used to separate harmonic and percussive from the mixed music, which was mainly used in the field of music signal processing. Compared with this kind of regularity and structured sound, the composition structure of environmental sound is more complex and usually contains non-harmonic and non-percussion sound segments. Hence, the HPSS algorithm proposed by Driedge^31^ is used to divide the audio signal into two parts, harmonic and percussive components. It will introduce a new type of separation factor to make the separated harmonics and percussive components more standardized.

**Figure 4:**
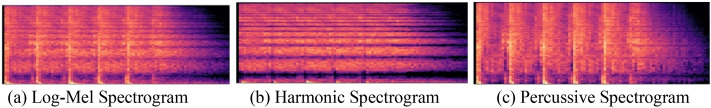
Harmonic Spectrograms and Percussive Spectrograms separated from the Log-Mel Spectrogram of the gunshot category using the HPSS algorithm.

In this paper, the HPSS algorithm is introduced to process the input Log-Mel spectrograms, which can be separated to obtain Harmonic Spectrograms and Percussive Spectrograms. In this way, the harmonic spectrogram can clearly illustrate the frequency distribution and frequency band activity of the audio data, shown in Fig. 4. In contrast, the impact component in percussive spectrograms has a very intuitive vertical structure, which can reflect the difference between the semantic related time frame part (gunshot) and other noise frames.

### Generate temporal-frequency attention mechanism

Environmental sound has complex time–frequency structure. In time structure, in addition to the semantic related time frame part, it also contains many silent or noisy parts. And since audio recording is usually in a polyphonic environment, there will inevitably be multiple sound sources, which makes it difficult to have a definite local relationship in the frequency domain. Therefore, the function of the proposed temporal attention mechanism is used to focus on the semantic related time frame part and suppress noise or silent frames. On the other hand, the frequency attention mechanism is introduced to assign more weight to the active frequency bands with distinguishing information, while to de-weighted for irrelevant frequency bands with less information.

**Figure 5:**
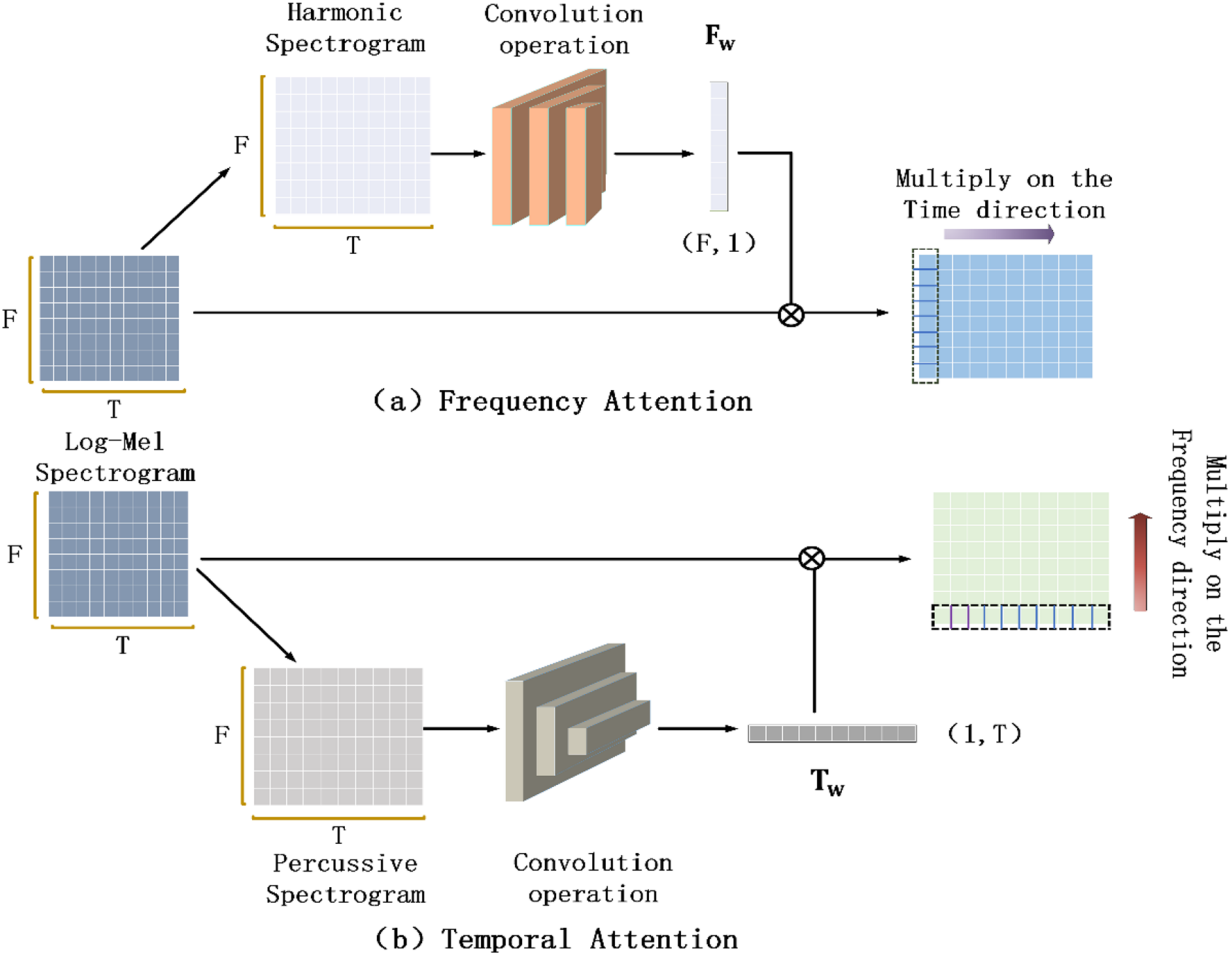
The generation process of two attention mechanisms.

As shown in Fig. 5, after standardizing the Log-Mel spectrogram **X**, the harmonic spectrogram and percussion spectrogram are separated by using the HPSS algorithm. Then, the convolution kernels with sizes of (1 × 3) and (5 × 1) are used to perform convolution operation on harmonic spectrograms and percussive spectrograms respectively to extract nonlinear features, until the time dimension of harmonic spectrograms and the frequency dimension of percussive spectrograms are reduced to 1, and then (1 × 1) convolution is used to compress channel information. In this way, two one-dimensional matrices ${\mathbf{A}}_{\mathbf{F}}$ and ${\mathbf{A}}_{\mathbf{T}}$ with sizes **(**$\mathbf{F}$**, 1)** and **(1, **$\mathbf{T}$**)** can be obtained. Finally, we use the Softmax function to normalize these two matrices to generate the frequency weight matrix ${\mathbf{F}}_{\mathbf{w}}$ and the temporal weight matrix ${\mathbf{T}}_{\mathbf{w}}$.

1 $${\text{F}}_{\text{w}}(\text{f})= \frac{\text{exp}({\text{A}}_{\text{F}}\left(\text{f}, 1\right))}{\sum_{\text{i}=1}^{\text{F}}\text{exp}({\text{A}}_{\text{F}}\left(\text{i}, 1\right))} , 1\le \text{f}\le \text{F}$$

2 $${\text{T}}_{\text{w}}(\text{t})= \frac{\text{exp}({\text{A}}_{\text{T}}\left(1,\text{t}\right))}{\sum_{\text{j}=1}^{\text{T}}\text{exp}({\text{A}}_{\text{T}}\left(1,\text{j}\right))} , 1\le \text{t}\le \text{T}$$

Next, the Log-Mel spectrogram **X** is point-multiplied with the obtained attention weight matrix in the time direction and the frequency direction respectively to obtain the frequency attention spectrogram ${\mathbf{S}}_{\mathbf{F}}$ and the temporal attention spectrogram ${{\text{S}}}_{{\text{T}}}$ , The expression is as follows:

3 $${\text{S}}_{\text{F}}(\text{f})=\text{X}\left(\text{F},\text{t}\right)*{\text{F}}_{\text{w}},\quad 1\le \text{t}\le \text{T}$$

4 $${\text{S}}_{\text{T}}(\text{t})=\text{X}\left(\text{f},\text{T}\right)*{\text{T}}_{\text{w}}, \quad1\le \text{f}\le \text{F}$$

Since the time and frequency domain of the spectrogram contains time and frequency feature information respectively, it is very different from the image in the visual classification task. The proposed two attention mechanisms can pay different attention to time frame and frequency band respectively, by combining the two mechanisms, the time and frequency features can be enhanced simultaneously to form complementary information. In general, the combination method is parallel or concatenation design, but the use of concatenation design to apply two kinds of attention to the spectrum in turn may cause the two mechanisms to interfere with each other, thus resulting in reduced system robustness. Therefore, this paper uses a parallel approach to design three combination strategies to combine the two mechanisms into a unified model.

The first strategy is referred to as average combination. Obtain frequency attention spectrogram ${\mathbf{S}}_{\mathbf{F}}$ and temporal attention spectrogram ${\mathbf{S}}_{\mathbf{T}}$ by applying two attention mechanisms to Log-Mel spectrogram. Next, the two attention spectrograms are fused into the final temporal-frequency attention spectrogram ${\mathbf{S}}_{\mathbf{T}\&\mathbf{F}}$ based on a 1:1 ratio. The specific operation process is as follows:

5 $${\text{S}}_{{{\text{T}}}}\& {\text{F}} {\text{Average}} = {\text{S}}_{{\text{T}}} + {\text{ S}}_{{\text{F}}}$$

The second strategy is referred to as weighted combination. Set up two learnable parameters ${\varvec{\upalpha}}$ and ${\varvec{\upbeta}}$ in the network, and limit them to ${\varvec{\upalpha}}+{\varvec{\upbeta}}=1$. The final temporal-frequency attention spectrogram ${\mathbf{S}}_{\mathbf{T}\&\mathbf{F}}$ is obtained by fusing the two attention spectrograms ${\mathbf{S}}_{\mathbf{F}}$ and ${\mathbf{S}}_{\mathbf{T}}$ according to the ratio of learnable parameters. The process can be expressed as:

6 $${\text{S}}_{{{\text{T}}\& {\text{F}}}} {\text{Weight}} = {\alpha S}_{{\text{T}}} + {\beta S}_{{\text{F}}}$$

The last strategy is referred to as channel combination. For the generated two attention spectrograms ${\mathbf{S}}_{\mathbf{F}}$ and ${\mathbf{S}}_{\mathbf{T}}$, concatenating them as two-channel output.

7 $${\text{S}}_{{{\text{T}}\& {\text{F}}}} {\text{Channel}} = {\text{joint}}\left( {{\text{ S}}_{{\text{T}}} { };{\text{ S}}_{{\text{F}}} { }} \right)$$

### Network architecture

The TFCNN architecture proposed in this paper consists of 6 convolutional layers, 3 pooling layers and 2 fully connected layers. Every two convolutional layers use the same parameters can be regarded as a block, and each block is accompanied by a max-pooling layer of size 2 × 2. The first two convolutional layers use 32 kernels with a size of 5 × 3, and the stride is set to 2. The kernel numbers of the remaining four convolutional layers are 64 and 128 respectively, the kernel size is 3 × 3, and the stride is 1. Finally, two fully connected layers with 256 hidden units are used on the flat output, and the output is further sent to the "Softmax" classifier to obtain the prediction result. In addition, the ReLU function is used as an activation function, batch normalization (BN) is used in each convolutional layer to speed up training, and a dropout mechanism is added to the fully connected layer with a probability of 0.5 to prevent overfitting.

### Decision strategy

**Figure 6:**
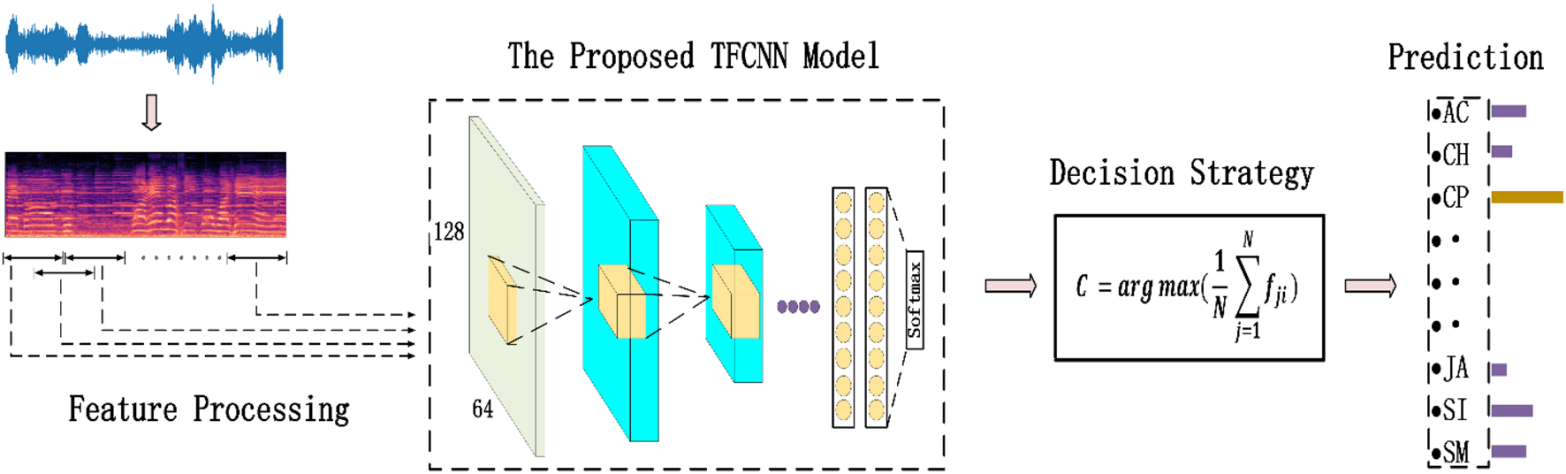
Probabilistic voting to predict the entire audio category.

In the process of feature processing, the log-Mel spectrogram divided into 64-frame sub-segments with a 50% overlap, and the label category of each sub-segment is consistent with the original audio. In the training phase, each sub-segment into the network for training, and predict the category for each sub-segment. In the final test phase, it is necessary to predict the entire audio category, and use the strategy of probabilistic voting to synthesize the predicted results of multiple sub-segments for judgment, as shown in Fig. 6. The mathematical expression is as follows:

8 $${\text{C}} = \arg \max \left( {\frac{1}{{\text{N}}}\sum\limits_{{{\text{j}} = 1}}^{{\text{N}}} {{\text{f}}_{{{\text{ji }}}} } } \right),1 \le {\text{i}} \le {\text{K}}$$

where, $\mathbf{N}$ represents the number of sub-segments divided into each audio sample, $\mathbf{K}$ represents the number of categories in the dataset, and $\mathbf{f}$ is the prediction result for each segment.

## Experiments and analysis

### Experiment setup

The research in this article was evaluated on the ESC-50 dataset^33^ and the UrbanSound8K^34^ dataset.

#### ESC-50

The ESC-50^33^ dataset consists of 50 different categories of audio data, mainly including: animals, natural soudscapes, water sounds, human non-speech sounds, internal/home sounds and external/urban sounds. Each category contains 40 audio data with a length of 5 s, totaling 2.8 h.

#### UrbanSound8K

The UrbanSound8K^34^ dataset contains 10 categories: air conditioner (AC,1000), car horn (CH,429), children playing (CP,1000), dog bark (DB,1000), drilling (DR,1000), engine idling (EI,1000), siren (SI,929), street music (SM,1000), jackhammer (JA,1000), gunshot (GS,374) contain a total of 8732 short audio clips (no more than 4 s), and the duration is about 7.3 h. Since the original audio is recorded at different sampling rates, a uniform sampling rate is first required. In addition, there is class imbalance in the dataset, making its generalization process rather difficult.

In this article, the Tensorflow framework is used, the development environment is Python 3.6.5, the hardware platform is NVIDIA GTX 1080, and Intel Core i7 CPU. The experiment adopted tenfold (UrbanSound8K) and fivefold (ESC-50) cross-validation strategies. Network training uses categorical cross entropy as the loss function, and uses the Adam optimizer for optimization. The learning rate, batch size and training epoch are set to 0.001, 100 and 200 respectively.

To evaluate the experimental results, this paper uses classification accuracy as a metric:

9 $$\text{Acc}=\frac{\text{the number of correctly classified}}{\text{Total number of test data}}$$

### Experimental analysis and visualization

To demonstrate the effectiveness of the methods proposed in this paper, we evaluated the baseline system and three combination strategies on the UrbanSound8K dataset. Based on the experimental results in Table 3, the following conclusions can be drawn: (1) Using the attention mechanism can indeed improve classification performance of the model. Compared with the model without the attention mechanism, the accuracy rate has been significantly improved. (2) The classification performance of the model after using the frequency attention mechanism is better than that of applying temporal attention mechanism. Considering the unstructured nature of environmental sound, it is obviously more effective to enhance the frequency features. (3) The proposed combination strategy has a further improvement in performance. Among the three combination strategies, the performance of weight combination is significantly better than the other two strategies. This illustrates that the time and frequency features of sound events do not play an equal role for model classification, but have certain emphasis.

**Table 3:** Classification accuracy after using different attention mechanisms.

Attention mchanism	ESC-50	UrbanSound8K
No attention	79.30%	90.77%
Temporal attention	81.90%	92.11%
Frequency attention	82.90%	92.34%
T-F attention(average)	83.80%	92.91%
T-F attention (weight)	84.40%	93.08%
T-F attention (channel)	82.20%	92.68%

We compare the attention mechanism generated by the spectrogram separated by the HPSS algorithm with that generated by the original log-Mel spectrogram. The experimental results are shown in Table 4. Although the two methods can improve the classification performance of the model, the harmonic spectrogram and the percussion spectrogram have clearer horizontal and vertical structure, the effect of promotion is better.

**Table 4:** Comparison of attention mechanisms generated by different spectrograms.

Dataset	Method	No attention	T-attention	F-attention
ESC-50	HPSS	79.30%	81.90%	82.90%
	Log-Mel		81.10%	82.40%
UrbanSound8K	HPSS	90.77%	92.11%	92.34%
	Log-Mel		91.65%	92.18%

**Figure 7:**
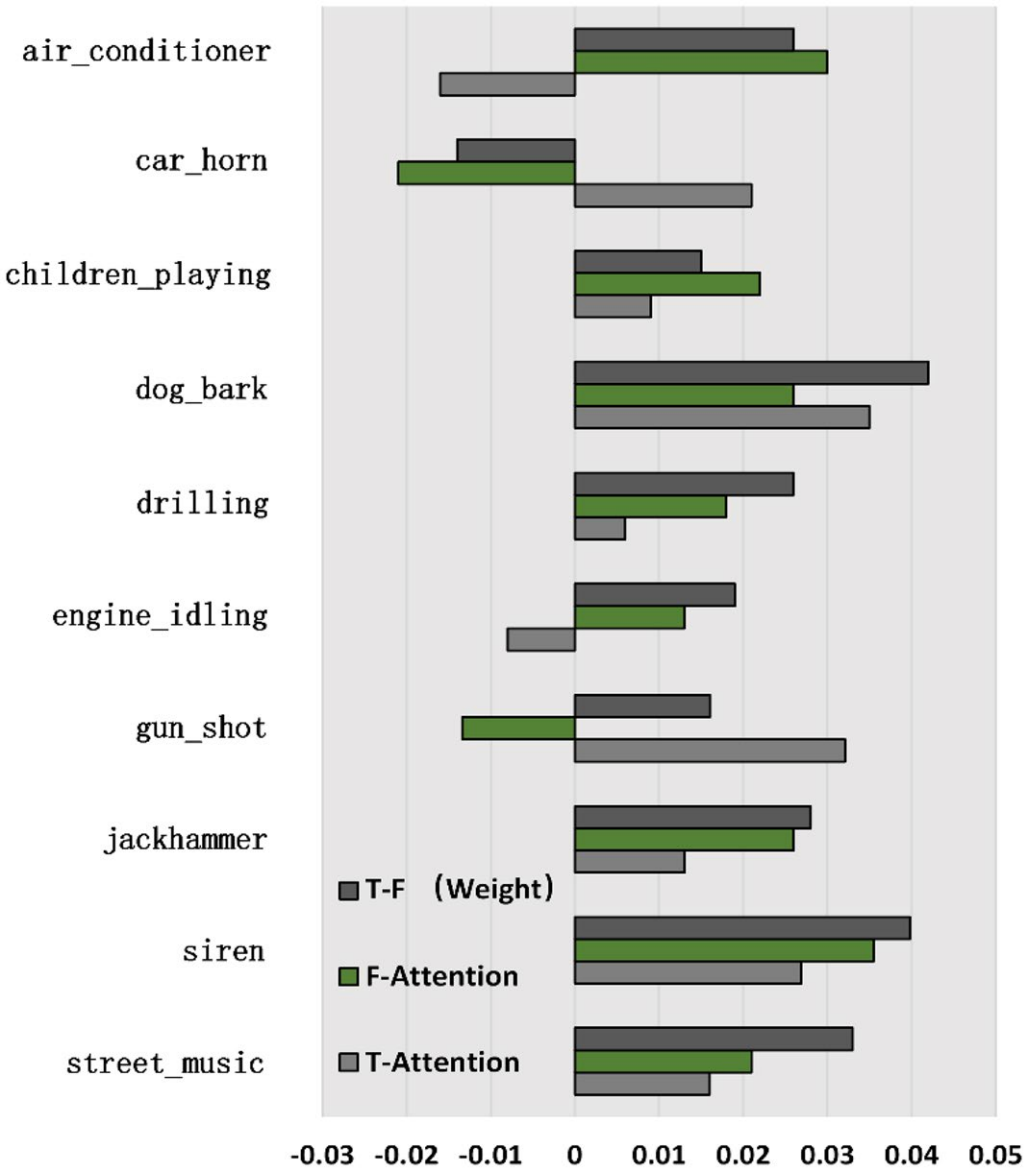
The difference of classification accuracy of each sound event after using different attention mechanisms on the UrbanSound8K datasets.

To further explore the impact of different attention mechanisms on the classification performance of the proposed model, Fig. 7 provides a difference of the classification accuracy of each sound event after using different attention mechanisms. It can be shown that after applying the attention mechanism, although the overall performance of the model has been significantly improved, for each sound event, the promotion effects of different attention mechanisms are not consistent. After using the temporal attention mechanism, it can greatly enhance the accuracy of transient sound, such as the "gunshot", "dog bark". For a frequency attention machine, the promotion effect is more obvious for continuous sounds such as "siren" and "air conditioner". This behavior is to be expected. For a transient sound, the semantic related time frame part in the audio sequence is usually discrete and contains a lot of silence and noise, while temporal attention mechanism focuses on specific time part, thereby reducing the influence of background noise on it. For continuous sound, the concentrated areas of active frequency bands become more prominent and have a strong distinction after applying frequency attention mechanism.

In addition, after using the weight combination strategy, the accuracy of most sound categories has been further improved, but there are still some categories ("air conditioner", "car horn", "children play" and "gunshot") that performance has not been enhanced and even have a negative impact. Considering that different sound events often have different time–frequency characteristics. This may mean that just setting a pair of learning parameters can lead to more outliers in individual categories, which is difficult to satisfy all categories.

**Figure 8:**
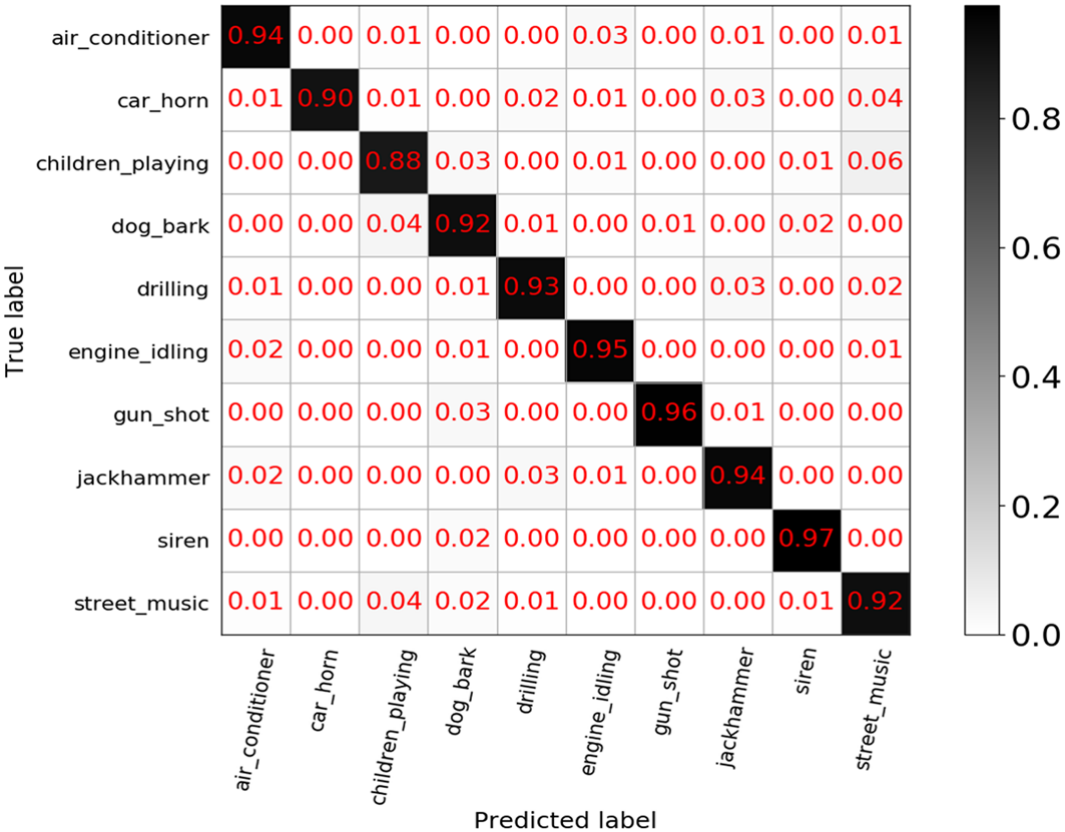
Confusion matrix for the classification accuracy of the proposed TFCNN model on UrbanSound8K datasets.

**Figure 9:**
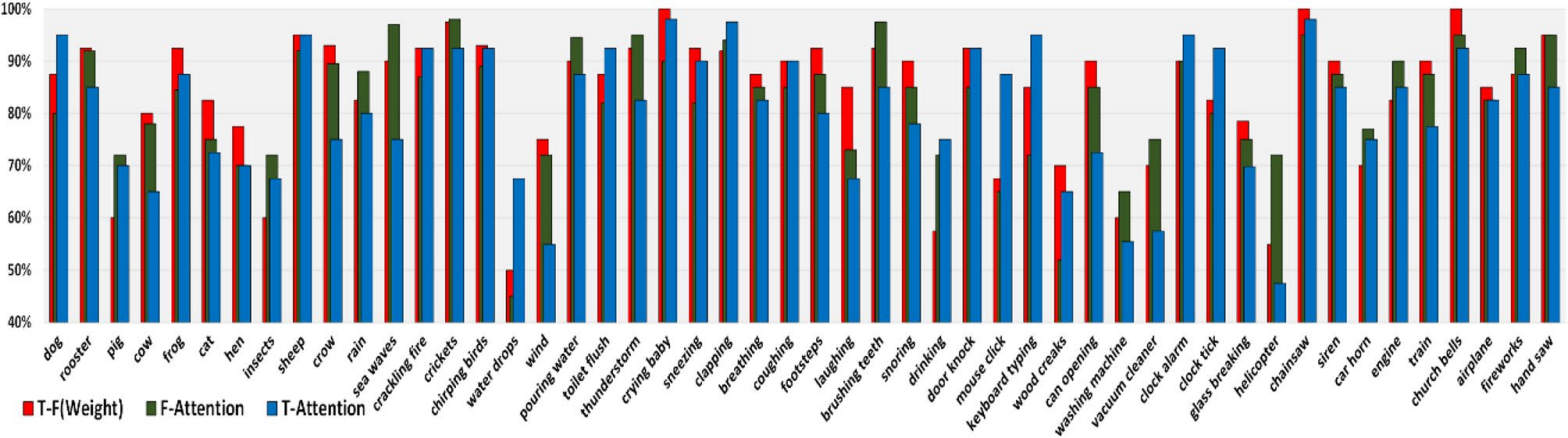
The classification accuracy of the TFCNN model on the ESC-50 datasets.

In Fig. 8, we provide the confusion matrix generated by the TFCNN model on the UrbanSound8K dataset. It shows that "children play" is the most difficult category to distinguish, and the categories of "siren" can be well recognized. In particular, "gunshot" and "car horn" are almost hard to be misclassified. Since the sample size of these two categories is much smaller than that of other categories, this phenomenon may be caused by the imbalance of categories. The classification results on the ESC-50 data set are shown in Fig. 9, TFCNN can achieve good performance in most categories, of which 37 categories have a classification accuracy of more than 80%, 22 categories are higher than or equal to 90%, only water drops and washing machine classification accuracy is not satisfactory, and 70% accuracy cannot be achieved under any attention mechanism.In addition, by comparing the accuracy of various categories under different attention mechanisms, it can be seen that most interior/domestic sounds and human (non-speech) sounds, such as "mouse click", "clock tick", "drinking" and "sneezing", belong to transient sound events, so they can achieve better performance when time attention mechanism is applied. For exterior/urban noises and natural soundscapes sounds, it is obvious that it can show high accuracy after frequency attention is applied because it contains most continuous sound events, such as "wind", "hand saw" and "helicopter". This result echoes the situation in the UrbanSound8k dataset, which once again illustrates the effectiveness and reliability of the attention mechanism.

**Figure 10:**
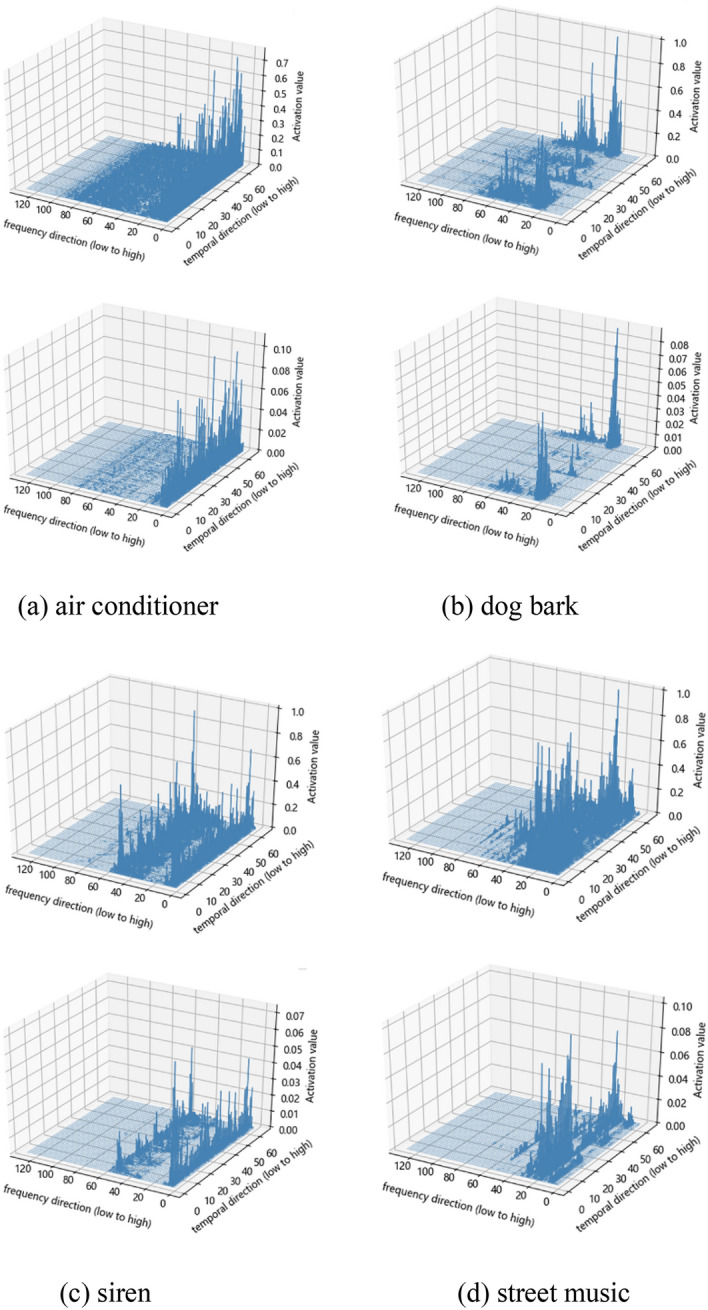
Visualize the feature distribution before and after the use of temporal-frequency attention for several sound events. The first row is the original feature distribution, and the second row is the attention-weighted feature distribution.

Figure 10 intuitively shows the changes in the features distribution before and after using temporal-frequency attention for several different sound events. It can be clearly seen that after using attention weighting, the time frame part and active frequency band containing more useful information get more attention, and the background noise and irrelevant frequency band are suppressed, so that the feature distribution of sound events becomes clearer and more distinguishable.

### Comparison to state-of-the-art methods

As shown in Table 5, the proposed model is compared with other models in the Urbansound8K and ESC-50 dataset.

1. Compared with single feature models: Piczak-CNN^19^, SB-CNN^26^, M18-CNN^27^,1D-CNN Gamma^28^, EnvNet2^32^, SoundNet^35^, Pyramid CNN^36^, DCNN^39^ all use a single feature representation. For Piczak-CNN^19^, SB-CNN^26^, Pyramid-Conmbined CNN^36^, DCNN^39^ use a two-dimensional feature map as input to extract deep features in a way similar to image classification tasks. EnvNet2^32^, M18-CNN^27^, SoundNet^35^ and 1D-CNN Gamma^28^ use the original waveform as input and extract feature from it. Compared with most of the methods described in the above-mentioned literature, the models proposed in this paper have achieved absolute improvements.
2. Compared with multi-feature models: DS-CNN^37^, M-LM-C CNN^38^, Two-Stream CNN^40^ and TSCNN-DS^41^ all use many different types of feature representations. M-LM-C CNN^38^ uses a single network architecture, which improves the classification performance of the model by providing more discriminatory and complementary feature representations. DS-CNN^37^, Two-Stream CNN^40^ and TSCNN-DS^41^ use ensemble models. Among them, DS-CNN^37^ and TSCNN-DS^41^ belong to the scoring ensemble, and they input different types of feature representations into two separate sub-networks for training, and finally ensemble the predicted results of each sub-network through DS evidence theory. Two-Stream CNN^40^ belongs to feature ensemble, the original audio and Log-Mel spectrogram is respectively input into two separate sub-networks of the model to extract feature representation, and then these features are merged to jointly train the model. As can be seen from Table 5, our model has better performance than DS-CNN^37^ and can compete with M-LM-C CNN^38^. Although compared with the best models such as Two-Stream CNN^40^ and TSCNN-DS^41^, the performance of the proposed models cannot be exceeded. But in addition to classification performance, another indicator for judging the pros and cons of the ESC method is the model complexity, which can be evaluated by comparing the number of trainable parameters. Considering that this paper uses a single feature representation and a single network architecture, and only uses a simple CNN to calculate the weight in the attention mechanism, the overall parameters are only slightly increased. While ensuring accuracy, it also has the advantages of low network structure complexity and simple feature processing, so our method is still competitive.

**Table 5:** Compare with other models on the Urbansound8K and ESC-50 dataset.

Model	Representation	Feature	ESC-50	UrbanSound8K	Parma
M18-CNN^26^	1D	RawData	–	71.7%	3.7 M
Piczak-CNN^19^	2D	Log-Mel	65.0%	73.7%	26 M
Pyramid CNN^36^	2D	Spectrogram	81.4%	78.1%	–
EnvNet2^31^	1D	RawData	81.6%	78.3%	18 M
SB-CNN^25^	2D	Log-Mel	–	79.0%	241 K
SoundNet^35^	1D	RawData	74.2%	–	–
1D-CNN Gamma^27^	1D	RawData	–	89.0%	550 K-
DS-CNN^37^	1D + 2D	RawData + Log-Mel	82.8%	92.2%	580 K
M-LM-C CNN^38^	2D	MFCC + Log-Mel + CST	85.6%	93.4%	11.3 M
DCNN(Augment)^39^	2D	Log-Mel	89.3%	95.4%	3.2 M
Two-Stream CNN^40^	1D + 2D	RawData + Log-Mel	–	95.8%	2.1 M
TSCNN-DS^41^	2D + 2D	Multiple Feature	–	97.2%	16.9 M
TFCNN (this paper)	2D	Log-Mel	84.4%	93.1%	1.6 M
Human^19^	–	–	81.3%	–	–

## Conclusion

In this paper, a new temporal-frequency attention based convolutional neural network model (TFCNN) is proposed for environmental sound classification. By introducing the developed temporal-frequency attention mechanism on the basic CNN architecture, the calculations used for representation learning can be concentrated on specific areas with discriminative information, thereby effectively capture critical time–frequency features. Experiments on the UrbanSound8K and ESC-50 dataset show that its classification accuracy is higher than 93.1% and 84.4%, respectively. Compared with the previous models on this dataset, our model has the advantages of low network structure complexity and simple feature processing while ensuring accuracy. In addition, this paper evaluates the classification performance of the model under several different attention mechanisms, and discusses their impact on each sound event. In the future work, we plan to continue to optimize the weighted combination strategy, according to the degree of dependence of different types of sound events on time and frequency features, and then selectively set the fusion parameters suitable for this category to further improve the performance of the model.
